# Genomic and transcriptomic resources for the tropical ascidian *Phallusia philippinensis*

**DOI:** 10.1093/g3journal/jkag057

**Published:** 2026-03-20

**Authors:** Shunsuke O Miyasaka, Takumi T Shito, Noburu Sensui, Euichi Hirose, Kotaro Oka, Kohji Hotta

**Affiliations:** Department of Bioscience and Informatics, Faculty of Science and Technology, Keio University, Yokohama 223-8522, Japan; Department of Bioscience and Informatics, Faculty of Science and Technology, Keio University, Yokohama 223-8522, Japan; Department of Chemistry, Biology and Marine Science, Faculty of Science, University of the Ryukyus, Okinawa 903-0213, Japan; Department of Human Biology and Anatomy, Graduate School of Medicine, University of the Ryukyus, Ginowan, Okinawa 901-2720, Japan; Department of Chemistry, Biology and Marine Science, Faculty of Science, University of the Ryukyus, Okinawa 903-0213, Japan; School of Frontier Engineering, Kitasato University, Sagamihara 252-0373, Japan; Department of Bioscience and Informatics, Faculty of Science and Technology, Keio University, Yokohama 223-8522, Japan

**Keywords:** tunicate, ascidian, *Phallusia philippinensis*, gene model, transcriptome, genome assembly

## Abstract

Due to remarkably stereotyped development and small numbers of cells, ascidian embryos are invaluable as models for investigating the principles of chordate development at single-cell resolution. Established model species of ascidians, such as *Ciona* spp. and *Phallusia mammillata*, are primarily distributed in temperate waters, and natural populations become scarce in the summer due to rising water temperatures. However, *Phallusia philippinensis* is a tropical/subtropical species that is closely related to *P. mammillata* and shares the feature of highly transparent embryos, which facilitates live imaging and optical analyses. Its accessibility in warmwater habitats makes *P. philippinensis* a promising alternative model for imaging-based analyses, particularly in tropical regions or during the summer. We present comprehensive genomic transcriptomic resources that have been resolved according to the developmental stage for *P. philippinensis*. The assembled genome spans 150.2 Mb and comprises 407 contigs with an N50 of 909 kb and a GC content of 42.0%. Transcriptomes from 5 organs and 9 distinct embryonic stages were used to construct a gene model, which revealed 20,928 genes with a high BUSCO completeness score of 88.6%. Functional annotations were assigned to 16,507 genes using the UniProt and RefSeq databases. We integrated these resources into a new constructed genomic browser, which can be used easily by all researchers. These new resources are immediately applicable to comparative developmental studies of gene expression and regulation in ascidian embryos and will comprehensively cover the off-season of reproduction for model ascidians.

## Introduction

Ascidians are a type of urochordate (tunicate), which is the sister group closest to vertebrates. Like vertebrates and cephalochordates (amphioxus), ascidians exhibit a canonical chordate body plan at the tadpole larval stage. Ascidian embryogenesis is simple and stereotyped with invariant lineages and a small number of cells (∼112 cells at gastrulation, which is consistent with other ascidian species, and ∼2,500 cells in the tadpole larva). This enables analyses of the developmental mechanisms at cellular resolution (Satoh 2014).

Species of the family Ascidiidae have unique advantages for bioimaging studies, including highly transparent gametes and embryos, as well as the ability to translate mRNAs in unfertilized eggs (Yasuo and McDougall 2018; Funakoshi et al. 2021). *Phallusia mammillata* is widely used in developmental studies (Robin et al. 2011; Yasuo and McDougall 2018), but its distribution is limited to Europe. *Ascidiella aspersa* is one candidate for use as an alternative in other regions. This species is also in Ascidiidae and native to Europe, although its distribution has expanded, and the species has become invasive in areas including Hokkaido in Northern Japan, where *P. mammillata* is not found (Lynch et al. 2016; Kanamori et al. 2017).

Recently, a new fertilization protocol and genomic resources have been developed for these species (Kogure and Hotta 2025; Shito et al. 2025). However, these 2 ascidians are temperate species, and collection of wild individuals is difficult during the summer due to rising water temperatures. Furthermore, there are no established model ascidians that are readily accessible to researchers in warmer regions. However, another Ascidiidae species, *Phallusia philippinensis*, inhabits tropical waters. This species occurs in tropical and subtropical habitats in the West Pacific, and its black tunic is thought to protect it from solar radiation (Hirose 1999). It has 2 similar congeners, *P. fumigata* and *P. nigra*, which have often led to taxonomic confusion (Vandepas et al. 2015).

*P. philippinensis* is commonly present on artificial structures in fishery ports and marinas in Okinawa, Japan. Due to its year-round availability of eggs and rapid early development, its embryos have been used in the field of developmental biology, particularly in research on fertilization (Sensui et al. 2012, 2023; Yoshida et al. 2013) and larval settlement (Sensui and Hirose 2020; Sakai et al. 2024). Hatching occurs only 12 to 13 h after insemination at 24 to 25 °C (Sakai et al. 2024). Despite these advantages, genomic and transcriptomic resources for this species are currently lacking. In this study, we established foundational genomic and transcriptomic resources for *P. philippinensis* and developed a web-based database with an intuitive user-friendly interface. These resources are expected to advance our understanding of its genetic features and to facilitate future research in developmental biology.

## Materials and methods

### Collection of organs and gametes

Adults of *P. philippinensis* were collected from shallow coastal breakwaters in the Ginowan Port Marina located on the west coast and the Yonabaru Marina on the east coast of Okinawajima Island (Okinawa Prefecture, Japan; June and October 2023). The animals were maintained at about 25 °C in an aquarium before experiments. Tunics were removed using scissors, and selected organs were dissected, immediately frozen with dry ice, transferred to sterile Eppendorf tubes, and stored at −30 °C until RNA extraction. Gonads and gonoducts were excised and subsequently washed with artificial seawater (ASW: Marine Art SF-1, Osaka Yakken) to dilute acidic body fluids. After blotting to remove residual fluids with Kimwipes, eggs and sperms were collected directly into sterile Eppendorf tubes by inserting a pipette into the oviducts and spermducts, respectively. For DNA extraction, sperm were immediately frozen with a dry ice and stored at −30 °C. Eggs were stored at 25 °C, and sperm-containing tubes were stored at 4 °C.

### Insemination and collection of embryos

Eggs were dechorionated using a mixture of 0.05% actinase-E and 1% mercaptoacetic acid sodium salt in ASW and then transferred into gelatin-coated plastic dishes filled with ASW. Sperm were preactivated with pH 9.5 ASW adjusted using CHES (2-(cyclohexylamino)ethanesulfonic acid) buffer, for 15 min before the insemination following Sensui et al. (2023). The eggs were washed with seawater 10 min after insemination. Embryos at several developmental stages were collected into microtubes, frozen using dry ice, and then stored at −30 °C until RNA extraction.

### DNA/RNA extraction and sequencing

For DNA extraction, frozen sperm were extracted from tubes and ground using a mortar and a pestle, and then refrozen using liquid nitrogen repeatedly. DNA extraction was performed in sperms dissected from 1 individual, using NucleoBond HMW DNA (Takara/Macherey-Nagel) which is suitable for yielding long DNA (up to ∼200 kb). Standard steps with supplied proteinase K/RNase A were followed. DNA quality was assessed with agarose gel electrophoresis; concentration and purity were measured by NanoDrop spectrophotometry and fluorescence-based dsDNA quantification Qubit Fluorometer (Thermo Fisher Scientific). Genomic DNA used for library preparation yielded 34.2 µg (234.0 ng/µl in 146 µl) with spectrophotometric purity ratios of 1.9 (260/280) and 2.2 (260/230) (Supplementary Table 1).

The following steps were performed by Takara Bio, Japan. Molecular-length distribution was checked on a pulsed-field capillary system (Agilent Femto Pulse System), confirming the main peak of DNA is about 10 kb containing DNA more than 100 kb. For library construction, genomic DNA was sheared to ∼20 kb with Megaruptor 3 (Diagenode) and processed using SMRTbell Prep Kit 3.0 (PacBio) for 3′ A-tailing, SMRTbell adapter ligation, and cleanup. Barcodes were added with the SMRTbell Barcoded Adapter Plate 3.0 (PacBio). After size verification, size selection was performed on BluePippin (Sage Science) with High-Pass Plus Gel Cassettes/Marker U1. Template preparation used the Revio polymerase kit (PacBio), and libraries were loaded onto Revio SMRT Cell trays/Revio sequencing plates and run on the PacBio Revio system. Data processing employed SMRT Link v13.1, generating CCS (HiFi) reads (QV ≥ 20) from ZMW multipass subreads.

For RNA extraction, frozen eggs and embryos were ground repeatedly. RNA Extraction was first attempted with the RNeasy Kit (QIAGEN). In some organs with difficulty of extraction, it was applied using a combination of TransZol Plant kit (TransGen Biotech) and NucleoSpin RNA Clean-Up kit (Takara). RNA quality was assessed with agarose gel electrophoresis and NanoDrop. All RNA samples exhibited high integrity (RIN values reported in Supplementary Table 4) and were used for stranded poly-A-selected library preparation. mRNA sequencing was performed using NovaSeq by Azenta (Japan). Poly-A selection was used to remove rRNA. Illumina NovaSeq produced 2 × 150 bp sequences for each sample.

### De novo assemblies of genome and transcriptome

PacBio DNA long reads (Revio HiFi) were assembled using 5 assembly software: wtdbg2 v2.5 (Ruan and Li 2020), SMARTdenovo (Liu et al. 2021), Hifiasm (Cheng et al. 2021), Flye v2.8.1 (Kolmogorov et al. 2019), and Canu v2.0 (Koren et al. 2017). The quality of assembly was assessed with SeqKit v2.10.1 (Shen et al. 2016) and BUSCO v5 (Manni et al. 2021). Repeat annotation was performed with RepeatModeler/RepeatMasker to aid soft masking during downstream analyses. Repeat sequences were identified using Repeat Modeler v2.0.4 (Flynn et al. 2020) and masked with RepeatMasker v4.1.5 (Smit et al. 2013 to 2015).

RNA sequence data were quality checked with FastQC v0.11.9 (Andrews 2010) and SeqKit. Adapter trimming was performed using Fastp (Chen et al. 2018). Adapter-trimmed reads were assembled using Trinity v2.14.0 (Haas et al. 2013). For polishing, adapter-trimmed reads were mapped to the genome assembly using Hisat2 (Kim et al. 2019), and almost more than 90% reads were aligned concordantly one time or more. Then only clean reads which mapped to the Trinity assembly were used for the following analysis. Cleaned RNA-seq pairs were aligned to the genome assembly with Hisat2, producing stage/tissue-wise BAM files that were sorted, indexed, and merged.

### Gene annotation and browser construction

For gene annotation, 2 software were used according to literature (Ito et al. 2024). BRAKER2 was used for an ab initio approach and used metazoan protein sequences (Brůna et al. 2021). As another approach, GeMoMa v1.9 (Keilwagen et al. 2019) was then used to extract RNA-derived intron evidence (module ERE) from the merged BAM, followed by CheckIntrons and DenoiseIntrons to remove low-coverage or abnormal introns. Homology-guided gene prediction was performed with GeMoMa using closely related ascidian references in combination with the intron evidence to generate transcript and protein models. Completeness of each model was assessed with BUSCO v5 using gVolante (Nishimura et al. 2017), and then the best combination of species was chosen. Gene models derived from BRAKER2 and GeMoMa were merged with GffCompare (Pertea and Pertea 2020). For functional annotation, EnTAP (Hart et al. 2020) was executed on the predicted proteins using protein resources: Universal Protein Resource (SwissProt, TrEMBL) (UniProt Consortium 2025) and RefSeq (Invertebrate) (Goldfarb et al. 2025). The transcription levels on each gene were counted by RSEM (Li and Dewey 2011).

## Results and discussion

### Genome sequencing and assembly

PacBio HiFi long-read sequencing (Revio HiFi) generated a total of 38.1 Gb across 2.31 million reads (SD0690_04_a), which corresponded to an estimated average coverage of 254× based on the 150.2-Mb assembled genome (Supplementary Table 1). Quality assessment indicated consistently high per-base quality scores (mostly above Q35), with per-sequence mean quality scores peaking at Q39 to Q40. The reads assembled using wtdbg2 (Ruan and Li 2020), SMARTdenovo (Liu et al. 2021), Hifiasm (Cheng et al. 2021), Flye (Kolmogorov et al. 2019), and Canu (Koren et al. 2017) yielded different assembly sizes of 207.3 Mb, 150.2 Mb, 1.44 Gb, 1.32 Gb, and 1.46 Gb, respectively (Supplementary Table 2). The numbers of contigs and corresponding N50 values were 3,826 (265 kb), 407 (909 kb), 7,171 (676 kb), 4,830 (640 kb), and 4,653 (1,389 kb), respectively.

Based on these results, the SMARTdenovo assembly was selected for downstream analysis. The final assembly of the *P. philippinensis* genome totaled 150.2 Mb across 407 contigs (mean 369.1 kb), with the largest contig of 6.9 Mb, an N50 of 909 kb, and a GC content of 42.0% (Table 1). The assessment of assembly completeness with BUSCO (Eukaryota *n* = 256) indicated 94.1% complete single-copy genes (single, 93.7%; duplicated, 0.4%), 2.0% fragmented, and 3.9% missing. Compared to genomes of related model ascidians, that of *P. philippinensis* has an intermediate size that is relatively compact (Supplementary Table 3). The contig count (407 contigs), N50 value, and BUSCO completeness also indicated moderate but sufficient assembly quality. These metrics confirm the attainment of a sufficiently contiguous and complete assembly that is suitable for downstream analyses.

**Table 1.** Statistics for the assemblies and annotations, including genome size, number of contigs, N50, BUSCO value, and number of annotations.

Genome assembly	
Genome size	150.2 Mb
Number of contigs	407
Mean length	369.1 kb
Largest contig	6.9 Mb
N50	909 kb
Repeat sequence	17.70%
GC (%)	42.0%
BUSCO complete	94.1%
Partial	2.0%
Fragment	3.9%
Gene model	
Genes	20,928
Transcripts	25,335
Annotation: with alignment	14,958
Annotation: with family assignment	16,014
BUSCO complete	88.60%
Partial	7.10%
Fragment	4.30%

### Transcriptome sequencing and assembly

**Fig. 1.**
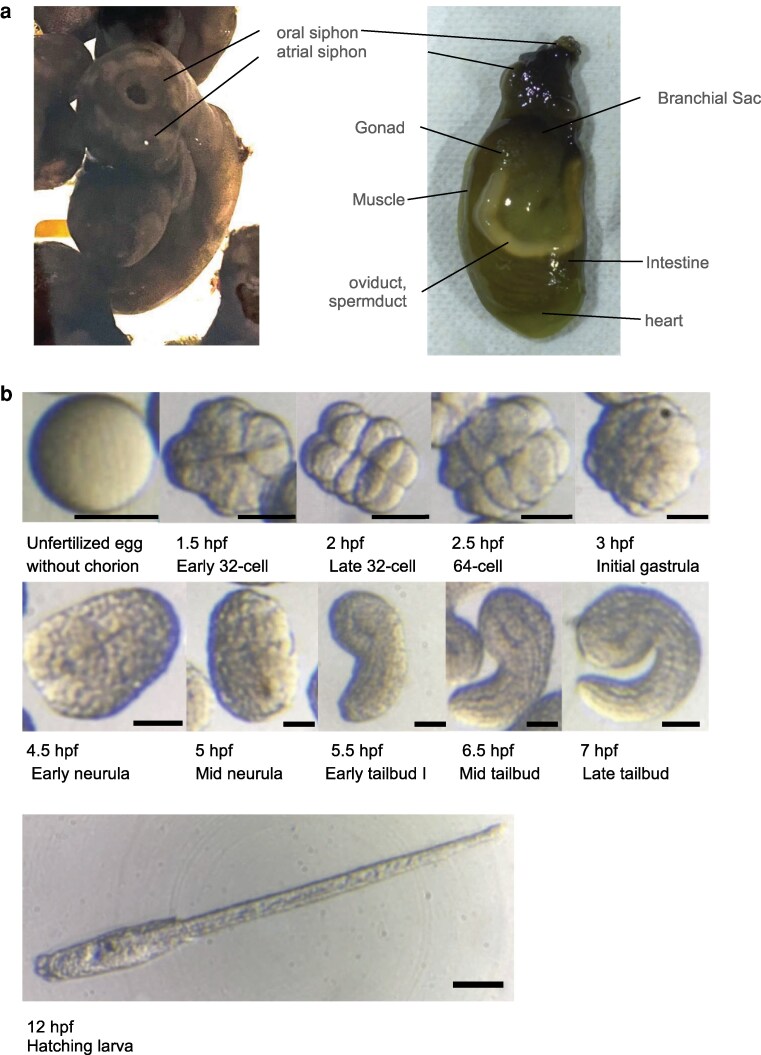
a) Body part of *P. philippinensis* (live specimen) used for RNA extraction. b) Embryos in each developmental stage used for RNA extraction. Scale bar:100 µm.

We successfully sequenced mRNA samples from 5 adult organs (branchial sac, heart, intestine, gonad, and muscle) and embryos from 9 different developmental stages from egg to hatching larva (unfertilized eggs, 32 cells, 64 cells, initial gastrula, mid-late gastrula, neurula, early tailbud, early-mid-late tailbud, and hatching larva; Fig. 1). Sequences were obtained using the Illumina NovaSeq platform. Following adapter removal and quality trimming, an average of 3.08 Gb of reads was generated in each case, which corresponded to approximately 21.6 million reads per sample (Supplementary Table 4). De novo transcriptome assembly with Trinity yielded 58,677 sequences with 74.3 Mb in each sample on average (Supplementary Table 5).

### Construction of gene models

BRAKER2 (Brůna et al. 2021) predicted 16,909 genes and 20,852 transcripts with a BUSCO completeness score 56.5%. BUSCO scores of 85.1%, 84.7%, and 85.9% were obtained from gene models generated using GeMoMa (Keilwagen et al. 2019) based on reference annotations from other ascidian model species: *Ciona intestinalis* Type A (*C. robusta*), *P. mammillata*, and *Ascidia mentula*, respectively (Supplementary Table 6). Among the tested combinations, gene models based on *C. robusta* and *A. mentula* showed the highest BUSCO completeness and were selected for further use.

Gene models derived from Braker2 and GeMoMa were merged using GffCompare (Pertea and Pertea 2020). The final gene model was designated as PP25 and contained 20,928 genes and 25,335 transcripts with a BUSCO completeness of 88.6% (Table 1; Supplementary Fig. 1). Functional annotation performed using EnTAP (Hart et al. 2020) resulted in functional assignments for 16,507 of the 20,928 predicted genes (Supplementary Table 7; Supplementary Fig. 1). A total of 14,958 of these predicted genes were annotated by similarity searches in major databases, including UniProt_SwissProt (8,501 hits), UniProt_TrEMBL (14,841 hits), and RefSeq_Invertebrate (11,003 hits). Gene Ontology (GO) terms were assigned to 13,476 genes, and KEGG pathway terms were associated with 5,021 genes (Supplementary Table 7).

### Online genome and annotation browser

Genomic and transcriptomic datasets can be difficult to navigate for experimental biologists who have limited experience in bioinformatics. To improve accessibility, we developed a user-friendly annotated genome and transcriptome browser for *P. philippinensis*. The platform includes an integrated BLAST interface that enables searches against either the assembled genome or the PP25 predicted gene models (Supplementary Fig. 2a). The browser includes functional annotation and supports searches by PP25 gene ID, human homologs, common gene names, motifs, and other keywords. Selecting a gene displays its expression levels (TPM) across adult organs and embryonic stages (Supplementary Fig. 2b and c). Both nucleotide and protein sequences are available on the same page.

The genome browser displays each predicted gene's genomic position together with mapped RNA-seq evidence using JBrowse (Diesh et al. 2023) (Supplementary Fig. 2d). The platform is fully web-based, accessible across devices, and freely available at https://ciona.bpni.bio.keio.ac.jp/PP25/Latest/Annotation.php. This database supports diverse experimental applications for *P. philippinensis*, including morpholino and sgRNA for CRISPR/Cas9 design, proteomic reference searches, and promoter-sequence retrieval for bioimaging.

Major model ascidians show distinct species-specific distributions, and no transparent species is accessible in all seasons or regions (Supplementary Table 3). As rising seawater temperatures further limit species availability during summer, access to warmwater models is increasingly important. With the genomic resources provided here, *P. philippinensis* could be a practical alternative model for developmental and functional studies in regions or seasons where other ascidians are not accessible.

## Conclusion

We have presented the first integrated genomic and transcriptomic resources for *P. philippinensis*, which comprise a 150.2-Mb high-contiguity assembly and stage-resolved RNA-seq that support well-annotated gene models. The datasets were integrated into a new genome and annotation browser that is freely accessible and user-friendly, particularly for experimental biologists. These resources are expected to facilitate multiomics analysis and functional studies in geographic regions or seasons where other model ascidians are not accessible.

## Supplementary Material

jkag057_Supplementary_Data

## Data Availability

The BioProject accession PRJNA1347562 provides access to the PacBio HiFi data, the genomic assembly Pphi_OKI2023, and all the raw RNA-seq data. The gene model set generated in this study is available in genome databases (https://ciona.bpni.bio.keio.ac.jp/PP25/Latest/Downloads.php). Supplemental material is available at G3 online.
