# Supplementary material for: Genomic and transcriptomic resources for the tropical ascidian Phallusia philippinensis
Source: G3 (Bethesda). 2026 Mar 20;16(5):jkag057. doi: 10.1093/g3journal/jkag057 (PMC13148405; doi:10.1093/g3journal/jkag057)

Supplementary Fig. S2. Genome and Annotation Database for *Phallusia philippinensis*

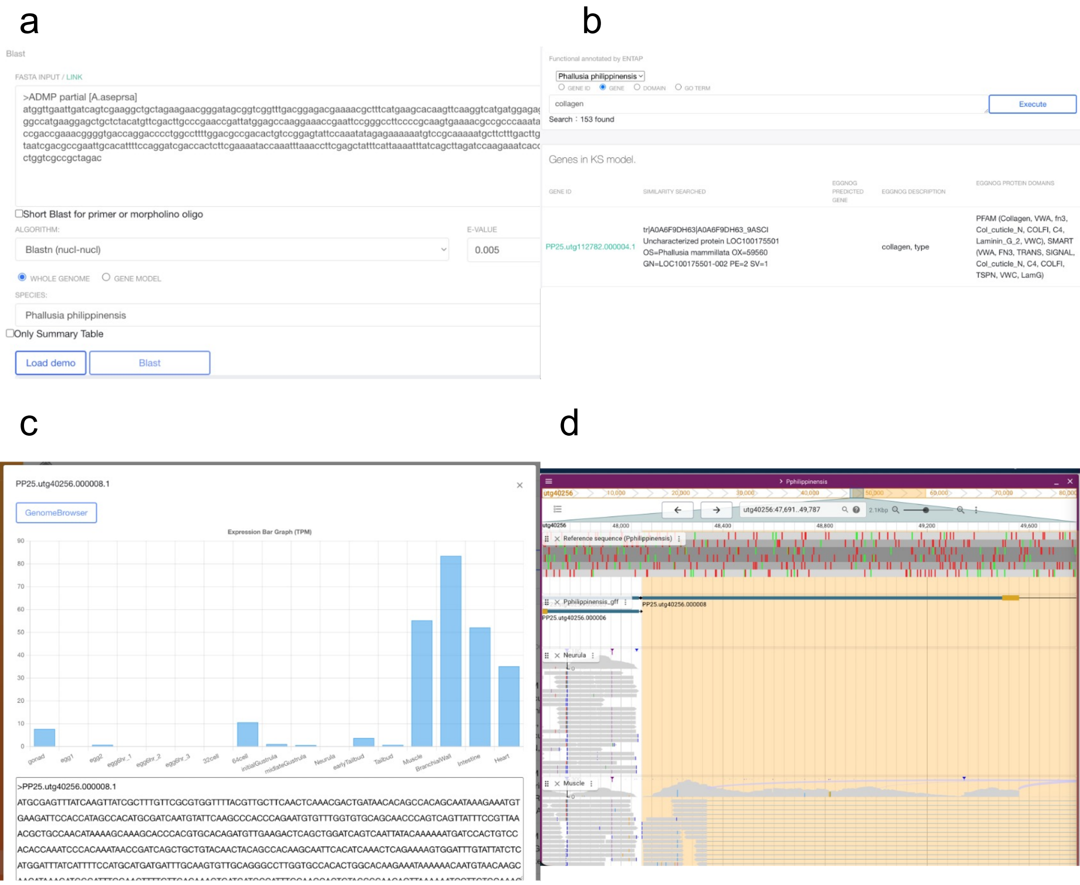

Supplement: jkag057_Supplementary_Data [file jkag057_supplementary_data.zip › Supplementary_Figure_S2_G3-2025-406443.pdf]
